# PageMan: An interactive ontology tool to generate, display, and annotate overview graphs for profiling experiments

**DOI:** 10.1186/1471-2105-7-535

**Published:** 2006-12-18

**Authors:** Björn Usadel, Axel Nagel, Dirk Steinhauser, Yves Gibon, Oliver E Bläsing, Henning Redestig, Nese Sreenivasulu, Leonard Krall, Matthew A Hannah, Fabien Poree, Alisdair R Fernie, Mark Stitt

**Affiliations:** 1 Max Planck Institute of Molecular Plant Physiology, Am Mühlenberg 1, 14476 Potsdam, Germany; 2 RZPD: Deutsches Ressourcenzentrum für Genomforschung GmbH, Heubnerweg 6, 14059 Berlin, Germany; 3 Leibniz Institute of Plant Genetics and Crop Plant Research (IPK), Corrensstr. 3, 06466 Gatersleben, Germany

## Abstract

**Background:**

Microarray technology has become a widely accepted and standardized tool in biology. The first microarray data analysis programs were developed to support pair-wise comparison. However, as microarray experiments have become more routine, large scale experiments have become more common, which investigate multiple time points or sets of mutants or transgenics. To extract biological information from such high-throughput expression data, it is necessary to develop efficient analytical platforms, which combine manually curated gene ontologies with efficient visualization and navigation tools. Currently, most tools focus on a few limited biological aspects, rather than offering a holistic, integrated analysis.

**Results:**

Here we introduce PageMan, a multiplatform, user-friendly, and stand-alone software tool that annotates, investigates, and condenses high-throughput microarray data in the context of functional ontologies. It includes a GUI tool to transform different ontologies into a suitable format, enabling the user to compare and choose between different ontologies. It is equipped with several statistical modules for data analysis, including over-representation analysis and Wilcoxon statistical testing. Results are exported in a graphical format for direct use, or for further editing in graphics programs.

PageMan provides a fast overview of single treatments, allows genome-level responses to be compared across several microarray experiments covering, for example, stress responses at multiple time points. This aids in searching for trait-specific changes in pathways using mutants or transgenics, analyzing development time-courses, and comparison between species. In a case study, we analyze the results of publicly available microarrays of multiple cold stress experiments using PageMan, and compare the results to a previously published meta-analysis.

PageMan offers a complete user's guide, a web-based over-representation analysis as well as a tutorial, and is freely available at .

**Conclusion:**

PageMan allows multiple microarray experiments to be efficiently condensed into a single page graphical display. The flexible interface allows data to be quickly and easily visualized, facilitating comparisons within experiments and to published experiments, thus enabling researchers to gain a rapid overview of the biological responses in the experiments.

## Background

Recent advances in microarray technologies have led to an avalanche of gene expression data from a variety of organisms. Many of these microarray experiments have been deposited in array databases and are available for public scrutinizing and data mining purposes [1-3]. Initially, microarray experiments involved comparison of one or a small number of treatments to a control. There is now a trend to more complex experiments, consisting of time-course (e.g. [4,5]) or dose-response studies. As an increasing number of data sets are deposited in the public domain, it is also becoming important to compare large numbers of treatments that are putatively similar, or that may share common components.

Given the initial limitation of only a few arrays per experiment, tools were first developed to visualize data on a one-experiment-at-a-time basis ([6-8]). Tools have also been developed that focus on the response of one (or several related) genes through multiple time points/experiments with a limited ontology structure [9]. There is still a need for visualization tools that allow data sets from multiple experimental conditions to be integrated and condensed into a single graphic display.

One common way to get an overview of a given microarray experiment is by using biological ontology structures. For example, many tools exist that utilize GO [10], KEGG, or MIPS functional categories to provide an overview of changes in expression using overrepresentation (ORA) Reviewed by [11] or other approaches [6,12]. As a complement to pre-defined annotations, *ad hoc *manual annotations or groupings of genes that are differentially expressed can be used for presentation and/or interpretation. In these approaches the functional ontology is often displayed as a tree-like graph, and the individual results are usually represented in tabular format.

In this paper we describe PageMan, a software tool which facilitates an ontologically-defined overview of the global response of the transcriptome. It provides a statistically-based overview of the enriched functional categories from global transcriptome responses, and can be viewed either in tabular form, or via a false color heat-map like display. This data-condensation allows the main global features of a single treatment to be rapidly identified, and facilitates the comparison of large numbers of treatments. PageMan can be used with various functional ontologies. It implements a Wilcoxon analysis to directly infer the contribution of individual categories to the response of the whole experiment [6]. It also implements an over/under representation analysis using either Fisher's exact test, or a χ^2 ^test combined with thresholds that the user can set individually. Furthermore, for convenience, a web based ORA analysis is offered on the PageMan website. PageMan generates direct-to-use, editable figures, which display both the hierarchy tree, as well as the changes within the individual experiments.

## Implementation

### Overview

PageMan is a standalone desktop application with Graphical User Interface (GUI) implemented in Java using the java swing libraries and parts of the MapMan source tree. Thus, PageMan should run on any Java enabled platform. It has been tested on Microsoft Windows XP, various Linux systems and on Apple's OS X operating system. Using a standard installation, PageMan can handle between 30–40 experiments at one time. However, by increasing the available java heap space to 1 GiByte enables PageMan to deal with hundreds of arrays.

The analysis algorithm for the Wilcoxon test has been described earlier [6]. For the ORA analysis a Fisher's exact or a χ^2 ^test is performed using a newly written Java class. The output of this class has been tested by performing the same tests in R [13]. This class can also be used independently of PageMan. In addition to the standard Java libraries, PageMan uses a number of third party libraries as support for specific operations. It relies on FreeHep libraries for graphical export, on JexcelApi library for import of excel files, which are one of the most widely used file formats of data representation in the biological sciences, and makes use of the Dom4j libraries for parsing XML files.

### Inputs

PageMan requires a mapping file, which assigns each probe identifier on the array into at least one functional category. In the examples presented here, the mapping file is based on the MapMan ontology for Arabidopsis genes described in the user's manual and elsewhere [6,14]. We provide a GUI tool to translate MIPS, KEGG, or GO hierarchies into this format thus enabling the user to choose the most appropriate ontology or compare the different ontologies.

PageMan can use several different types of input for the experimental data, depending on the operation desired e.g. log_2 _fold change or p-values of differential expression obtained by freely available, standard array handling software such as BioConductor [15]. Alternatively, if the user requires only data visualization it is possible to generate a PageMan native file (in tab separated text format, see user's manual for details). Using this format it is possible to display any kind of data as false-color boxes, thus enabling the use of PageMan for a multitude of other applications.

### Computation within PageMan

#### Over/Under-representation analysis

For over-representation analysis, PageMan uses Fisher's exact or a χ^2 ^test to calculate the likelihood for each category to contain the number of objects exceeding a user-definable threshold that is actually observed, given the total number of objects and the total number of objects exceeding this threshold. This threshold depends on an analysis previously performed. For example, one could analyze which genes surpass a certain fold-change value, or which genes are below a certain p-value, if differential expression has already been calculated. To facilitate interpretation, PageMan applies the same procedure for objects below the negative value of the threshold and adds the tags "up" or "down" to the experiment names respectively.

Unlike the χ^2 ^test, Fisher's exact test is also applicable for extreme cases of test situations, such as only observing a small number of objects per class or small classes. However, a χ^2 ^test with Yates continuity correction is offered as an alternative for testing; in this a case, ontological groups with too few items are omitted. The calculations are based upon the approximation of the Gamma function by Lanczos [16] as implemented in the Gnu Scientific Library which has been ported to Java [17].

#### Wilcoxon Test Statistic

PageMan uses an internal routine to compute an unpaired Wilcoxon rank sum test statistic (equivalent to Mann-Whitney's U test). If a table of fold-change values was given as an input, this feature would test whether the median fold-change within a particular ontological group was the same as the median fold-change of all genes not in that ontological group. Unlike ORA based tests, the Wilcoxon test does not require setting a sometimes subjective threshold.

#### Multiple hypothesis testing correction

PageMan allows the user to not only test one hypothesis (e.g. is glycolysis up-regulated/over-represented?) but to test up to hundreds of hypotheses at once (are any of the functional categories changed?). It is therefore necessary to implement multiple-hypothesis-testing correction methods. This is achieved using three different methods: the conservative Bonferroni correction that controls the family wise error rate, and the false discovery rate control methods by Benjamini, Hochberg [18] and Benjamini,Yekutieli [19]. After correcting for multiple testing, "adjusted" p-values are computed according to the correction method specified. In the case of the false discovery rate controlling corrections, these new values actually represent the false discovery rate level e.g. using a value of 0.05 as a cut-off would mean accepting a false discovery rate level of 5%. None of these testing corrections takes the nested hierarchy into account and may therefore lead to slightly biased results.

#### Conversion for display

In order to display (adjusted) p-values in PageMan, they are transformed into their respective z-values. All p-values above 0.05 are set to a z-value of 0 to avoid misinterpretation. The resulting values are then false color coded in a user-adjustable two color scale. Here, a highly saturated color indicates a high absolute value, whereas smaller values are indicated by a lower color saturation. For the Wilcoxon's test p-values, two different colors (e.g., blue and red) can be selected to distinguish between categories where the average of the signals for all the genes in a category increases or decreases.

### The PageMan GUI

**Figure 1:**
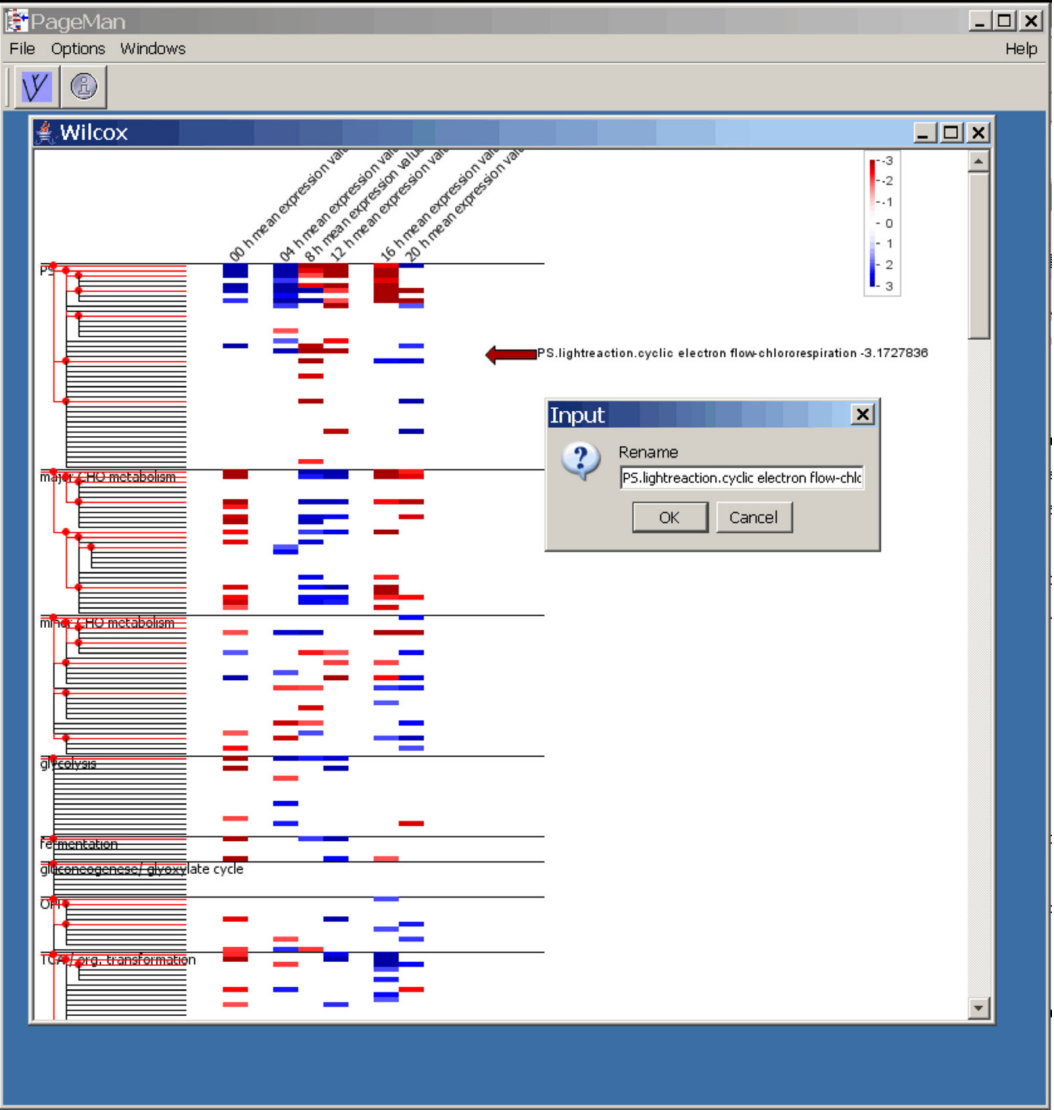
**PageMan Analysis and Annotation Session**. An ongoing annotation session. The user is presented with the results of the statistical analysis using a false color scale. On the left hand side, the user can access an ontology tree which can be collapsed on demand. On the right hand side a color scale is shown.

PageMan was designed with ease of use in mind: for this reason the user is guided through the analysis by a wizard. Once the analysis has finished, the user is presented with a heat map (overview transcript map) with representation of the differently enriched/differently behaving functional categories within the various experiments by false color coded boxes. This view can be overlaid with a tree representing the hierarchical information among different functional categories (Figure 1, left hand side). For flexible visualization, individual nodes of the hierarchy tree can be collapsed to remove areas of the tree that are uninteresting (e.g. because there are non-significant changes). Alternatively, all parents having only non-significant nodes can be collapsed or all non-significant nodes can be hidden. The boxes of the false color display can be identified and annotated by clicking or by using the command to "annotate all significant nodes"; this allows an editable, moveable annotation arrow to be added directly opposite the heat map feature. Annotations that are not required can be manually removed. Finally, experiments can be deleted from the display, and spacers can be added to separate groups of experiments to optimize the visual appearance.

For the layout, several different options are accessible via the options menu. For example, the dimensions of the boxes as well as the color intensities for the boxes can be set according to the user's choice. For depicting differential expression, several different color schemes (red-white-blue, red-black-green etc.) are available. Finally, sub-categories can be opened in a separate window for closer inspection.

### Graphical Output

PageMan comes with various graphical export capabilities, which support the production of suitable graphics for viewing or even for pre-publication stages. The visualization display can be exported in standard bitmapped formats (such as png or jpeg), and in vector formats such as svg, ps, pdf and the windows specific emf format. This allows the visualization to be imported into various downstream applications such as Microsoft PowerPoint or Corel Draw, where the individual elements can be further edited without loss of quality for final manuscript preparation and/or presentation. As indicated above, it is possible in advance to collapse or expand nodes while preparing the visualization display, in order to focus on selected features of the response.

### Documentation

Help is available directly from the program itself by simply selecting the help menu item from within PageMan. Moreover, on our website we offer a step-by-step tutorial that guides one through the use of PageMan.

## Results and discussion

### Exemplary comparison of multiple experiments

**Figure 2:**
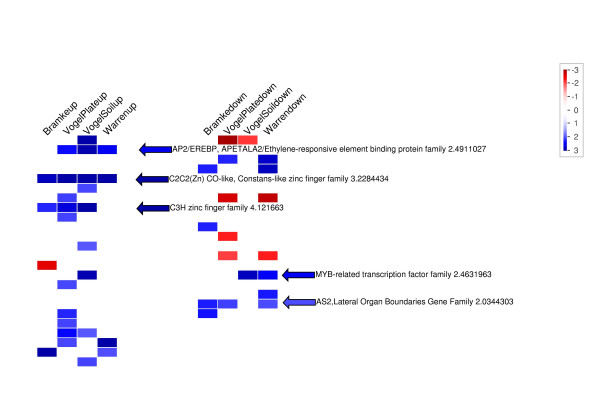
**PageMan Analysis of a Cold Stress Time Series**. Here, different cold stress experiments obtained from NASC were supplied as log_2 _fold change values. An over-representation analysis was performed using Fisher's exact test and 1 as a cut-off. Using PageMan's "zoom in" function, only potential transcription factors were displayed.

To demonstrate the use of PageMan for multiple experiments, Arabidopsis cold stress experiments were downloaded from NASC Arrays [3] and evaluated. The RMA expression values for the samples were calculated [20] and a linear model was fitted using BioConductor [15,21]. The resulting log_2 _fold change values at each time point were calculated and used for PageMan. The data was processed in PageMan using ORA analysis with Fisher's exact test, setting a threshold of 1 (at least a two fold change). All categories that have more/less genes than expected that exceed this threshold are colored with increasing intensity. An example from the PageMan visualization is shown in figure 2, where categories for transcription factors have been magnified using PageMan's "extract and enlarge" function. AP2/EREBP and Constans-like transcription factors are consistently over-represented amongst the up-regulated genes. Over-representation of MYB related genes amongst the down-regulated genes can be seen in most experiments. These responses are in accordance with earlier meta-analysis of cold acclimation using MapMan ontologies performed by Hannah et al. 2005 (see Table S6 from Hannah et al. 2005[22]). However, the earlier analysis was time-consuming, requiring either manual bin counts or scripting based on customized mapping files. PageMan performs this type of analysis in a few minutes, including annotation and layout, resulting in a graph like that shown in figure 1. It also allows equally rapid analysis using the other three enrichment-based statistical tests included in the package. Thus, PageMan provides a quick integration at the ontology level across multiple similar experiments, and allows comparison of their similarities and differences. As exemplified, the PageMan graphical interface provides an intuitive visualization overview representing "hot-spot pathways" activated during Arabidopsis cold stress across experiments performed by different labs.

### Comparison of PageMan with related tools

Most currently available tools are limited to a few (usually enrichment-based) statistical models such as either the hypergeometric or the binomial distribution. Within the 14 tools reviewed by Khatri and Draghici, only one, namely the Onto-Express tool, supported four different enrichment-based statistical tests. PageMan supports the use of Fisher's exact test and χ^2 ^statistics as well as Wilcoxon's test. Unlike the web-based tool JProGO [23] it allows use of the non-enrichment based Wilcoxon test, without the web-based limitations. Also, many tools are limited to a few experiments at a time, whereas using PageMan evaluating hundreds of experiments at once is possible.

Most available tools only support the GO ontology, or GO and KEGG in the case of AMDA. To the best of our knowledge, PageMan is the only tool supporting the use of MapMan, KEGG, MIPS, and GO ontologies. By providing a parser to automatically format these ontologies, PageMan offers the user unprecedented flexibility to use whatever ontology is strongest or most advanced in their particular field of study. Thus using PageMan it is also possible to classify metabolite data, which is not possible based on the GO ontology. As discussed by Khatri and Draghici [11], most tools that use the GO ontology are not able to use a higher level of abstraction because they can only use the lowest level of the hierarchy. PageMan allows the user to flexibly collapse nodes that are of no interest for the user, and by default analyze all levels of abstraction.

Also, many tools are limited to a few experiments at a time, whereas using PageMan evaluating hundreds of experiments at once is possible. Further, PageMan supports the subsequent introduction of more array data (including that from a different organism or a different array platform) for comparison. Among the tools having a user interface, this represents a rather novel feature. Although this is also possible by using R/Bioconductor, substantial programming skills are required. As Manoli et al. point out, group testing helps in comparing different datasets [24].

In terms of graphical capabilities and the ability to upload multiple experiments, High-Throughput GO-Miner [25] and AMDA [26] are most similar to PageMan, as they also offer heat-map like graphics. However, unlike PageMan, these tools are not interactive. High-Throughput Go-Miner sometimes requires manual editing of configuration files, and the installation requires connecting to an SQL server or for the user to install their own SQL server. This is typically beyond the means of most users. Further, although a database approach offers more flexibility, the use of dumped files for classification (as in PageMan) offers a significant speed improvement because the time intensive step of connecting to a remote database or a web-service only needs to happen once. AMDA, while offering a widget based interface still requires the installation of Tcl/Tk on top of R and BioConductor, which can be cumbersome on Windows. PageMan, on the other hand, is packaged with an installer and the user can download necessary files from the internet and thus remain totally anonymous and independent of internet connections for analyses. Further, unlike PageMan, the heat-maps generated by High-Through Put GO Miner or AMDA are static and annotations can not be easily edited.

## Conclusion

We have developed a novel, platform-independent tool, PageMan, which is available free of charge. It aids in interpreting individual microarray experiments and in exploring large sets of microarray experiments by analyzing and summarizing the data, and then visualizing it in an ontological context. With this tool it is possible to quickly compare given data to published results and/or to pinpoint special biological processes or pathways that may need to be investigated more thoroughly. PageMan also allows comparison of the global response to analogous treatments in different species, provided that a comparable ontology is possible (see e.g. [27] and [28], for an extension of the MapMan ontology to tomato and *Medicago*). It is planned to extend the MapMan ontology to further crop plants and wild plant species, as large scale array sets become available for them. Moreover, PageMan will be adapted to include p-value corrections that take nested hierarchies into account, once these become widely accepted.

Even though many tools have been generated over the years that use ontological categories to statistically assess and summarize data, PageMan offers the unique possibility to layout, visualize, and annotate information from large transcriptome series experiments in an integrated manner using a single tool. Furthermore, it is generic, and can be applied to other large quantitative data sets obtained from enzymatic, metabolomics, or proteomic approaches. This offers the research community a tool to both globally analyze and identify "hot-spot regulated pathways" and immediately export publication ready pictures.

## Availability and requirements

• Project name: PageMan

• Project home page: 

• Operating system(s): Platform independent

• Programming language: Java

• Other requirements: Java 1.4 or higher

• License: freely available. The software uses libraries covered by the LGPL (freehep for graphics import) and others (dom4j for xml import).

• Any restrictions to use by non-academics: none

## Authors' contributions

BU designed the software, implemented the visualization, and tested and improved the statistics. AN implemented data loading, foreign ontology parsing, document handling as well as parts of the user interfaces, AN streamlined the whole code, and fixed bugs. MS presented the problem and came up with an initial concept, discussed additional users' needs and presented UI improvements. HR identified the Wilcoxon test as a possible tool and MH tested and discussed ORA analysis. YG, OB, and MS brought up the concept of data condensation. FP discussed UI improvements. ARF contributed tomato ontologies, NS contributed barley ontologies. YG, OB, ARF, NS, DS, HR, LK, FP and MS located mistakes in the software. All authors have read and approved the final manuscript.
